# Optical Coherence Tomography Angiography Findings in Primary Progressive Multiple Sclerosis Patients Receiving Ocrelizumab Treatment

**DOI:** 10.3390/diagnostics16060936

**Published:** 2026-03-22

**Authors:** Burçin Çakır, Seren Kaplan Güngördü, Nilgün Özkan Aksoy, Dilcan Kotan

**Affiliations:** 1 Department of Ophthalmology, Faculty of Medicine, Sakarya University, Sakarya 54187, Turkey; 2 Department of Neurology, Faculty of Medicine, Sakarya University, Sakarya 54187, Turkey

**Keywords:** primary progressive multiple sclerosis, macular vascular density, retinal nerve fiber layer thickness, optic neuritis

## Abstract

**Objectives**: The aim of this study was to evaluate macular vessel area densities (superficial and deep) and foveal avascular zone (FAZ) measurements using OCT-A in the eyes of primary progressive multiple sclerosis (PPMS) patients receiving Ocrelizumab treatment with or without optic nerve involvement. **Methods**: The medical records of PPMS patients who received Ocrelizumab treatment at least once were reviewed. Retinal nerve fiber layer (RNFL) thickness measurements and OCT-A analysis were conducted on the PPMS patients and on age-matched healthy individuals. The patient group was divided into two subgroups: eyes with optic neuritis (PPMS+ON) and eyes without ON (PPMS-ON). Central and mean superficial vessel area (SVA) and deep vessel area (DVA) densities, as well as foveal avascular zone (FAZ) measurements, were analyzed. All parameters were statistically compared between groups and subgroups. **Results**: A total of 38 PPMS patients receiving Ocrelizumab treatment and 31 healthy individuals were included in this study. Statistically significant differences were observed between the groups in terms of best corrected visual acuity (BCVA), RNFL thickness, and the superficial vessel area densities for all parts except for the central part. In terms of deep vessel area densities, differences were found in the central and inferior parts. The mean FAZ area also showed a statistically significant difference between groups. Mean RNFL thickness differed significantly between the subgroups. Mean nasal, temporal, inferior part, and total superficial vessel area densities were statistically different between the subgroups. The central and inferior parts of the deep vessel area densities showed statistically significant differences. The mean FAZ area was also statistically different between the subgroups. **Conclusions**: The findings suggest that macular superficial and deep vascular densities are affected in PPMS patients receiving the same therapy modality and that previous optic neuritis may influence the results.

## 1. Introduction

Multiple sclerosis (MS) is an inflammatory autoimmune disease that causes demyelination and axonal loss in the central nervous system (CNS). The disease follows four clinical courses: relapsing–remitting MS (RRMS), clinically isolated syndrome (CIS), secondary progressive MS (SPMS), and primary progressive MS (PPMS) [1]. PPMS is characterized by a progressive deterioration of symptoms from commencement, lacking identifiable relapses or remissions [2].

The treatment options for patients with progressive forms of MS are generally limited, although the recent availability of novel treatments has expanded the disease-modifying treatment options for these MS phenotypes. Ocrelizumab is the only disease-modifying drug approved for the treatment of PPMS and has been proven to slow down disability progression and brain volume loss [3,4,5].

Optical coherence tomography (OCT) has been widely used to evaluate retinal nerve fiber layer (RNFL) and ganglion cell and inner plexiform layer (GCIP) measurements in patients with all types of MS, including PPMS [3,6,7]. Neuroaxonal injury can be detected through OCT and measured in terms of decreases in RNFL and GCIP thickness relative to that in normal control subjects. Furthermore, evidence of CNS inflammation may be found in the form of inner nuclear layer thickening, potentially due to the formation of microcystic macular edema [6].

Aside from immune-based etiologies of MS, increasing attention is being paid to discovering possible vascular elements contributing to the pathogenesis of this disorder [8]. Optical coherence tomography (OCT) and OCT angiography (OCT-A) allow for non-invasive, high-resolution imaging of retinal blood vessels [9,10,11]. Rarefaction of the superficial vascular complex (SVC) assessed by OCT-A, which supplies blood to the RNFL and GCIP, is evident in eyes with and without a history of optic neuritis and has been found to be associated with worse visual function, disability, and prognosis in patients with MS [12].

Currently, no study in the literature has investigated macular vessel area densities in PPMS patients receiving Ocrelizumab treatment. Therefore, the aim of the current study is to evaluate macular vessel area densities (superficial and deep) and the foveal avascular zone (FAZ) measured by OCT-A in the eyes of PPMS patients receiving Ocrelizumab treatment and to determine the possible effect of optic nerve involvement.

## 2. Material and Methods

This cross-sectional retrospective study was conducted at the Ophthalmology and Neurology Departments. Prior approval from the Ethics Committee of Sakarya University (IRB number: E-71522473/050.01.04-275931-253, date: 25 August 2023) was obtained, and written informed consent was acquired from each subject. This study was conducted in adherence to the Declaration of Helsinki.

Records of patients with multiple sclerosis who were followed at the Neurology and Ophthalmology Clinics between 2022 and 2024 were reviewed, and those with primary progressive multiple sclerosis who had received at least one course of Ocrelizumab treatment were selected. Measurements were taken before their next Ocrelizumab therapy. A diagnosis of MS was made at the Neurology Clinic according to the 2010 McDonald criteria. Patients’ MS disability levels were evaluated based on their expanded disability status scale (EDSS) scores and patients with 2–3 EDSS scores were recruited. PPMS patients who underwent RNFL thickness measurements and OCT-A analysis formed the patient group. The patient group was classified into two subgroups: eyes with optic neuritis (PPMS+ON) and without ON (PPMS-ON). Patients with any other systemic diseases were excluded. Age- and sex-matched healthy individuals without systemic and ocular diseases who applied consecutively and underwent RNFL thickness and OCT-A analysis formed the control group. For both groups, all types of eye diseases causing a decrease in visual acuity were assessed as exclusion criteria, including history of ocular surgeries, retinal diseases, hyperopia and myopia higher than 3 diopters, astigmatism higher than 2 diopters, and all types of cataracts affecting the quality of OCT and OCT-A tests. Patients who had an ON attack within 6 months of the measurements were also excluded.

The duration of PPMS, number of Ocrelizumab infusions, age, and gender were noted. Best corrected visual acuity (BCVA) and history of optic neuritis were also documented. A diagnosis of optic neuritis was made based on ophthalmological examination findings, including visual acuity, color vision, visual field test, optic disk evaluation, light reflexes, and visual evoked potentials (VEPs). An additional MOG-Ig-G serum test was performed for the differential diagnosis.

**Figure 1:**
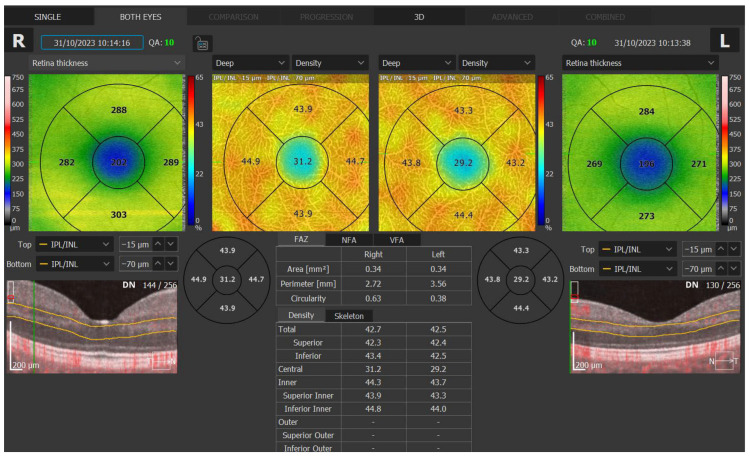
Optical coherence tomography angiography macular vessel area density measurement.

**Figure 2:**
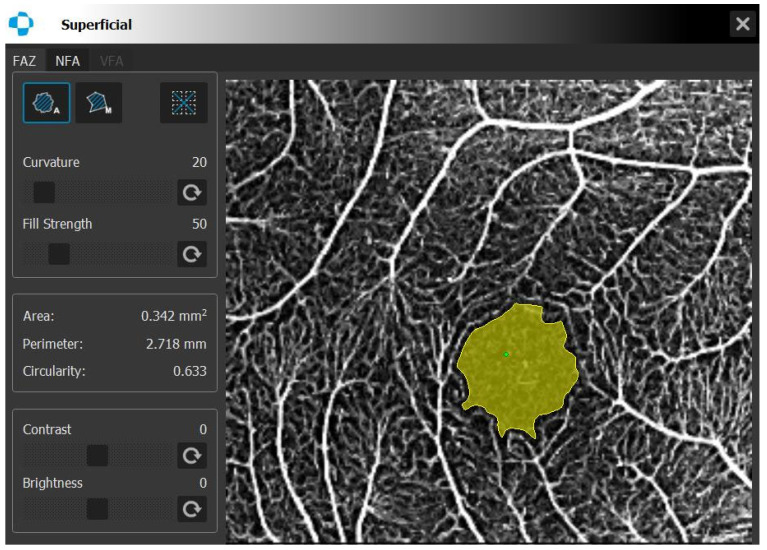
Optical coherence tomography angiography foveal avascular zone measurement.

Central and mean superficial (SVA) and deep vessel area (DVA) densities and foveal avascular zone (FAZ) measurements were performed using Spectral domain OCT-A (Optopol Technology, REVO FC 130, Software version 11.5.1, Zawiercie, Poland). The Angio scan constructed from angiography OCTA data was acquired from 230 A-scans and 230 B-scans as the default for REVO. In REVO nx, the operator can modify the scanning protocol with a maximum resolution of 512 A-scan and 512 B-scan. The Angio scan constructed from angiography OCT-A data was acquired from 320 A-scans and 320 B-scans as the default for REVO FC. The retinal layer boundaries were automatically drawn but could be customized by the user. The SVA densities were measured between the outer boundaries of the outer and inner plexiform layers. The DVA densities were measured between the outer boundary of the inner plexiform layer and the inner boundary of the nerve fiber layer. In the macular zone, OCT-A was performed within a 3 × 3 mm (256 × 256) area. Vessel area densities at the superficial and deep levels in four quadrants (superior, inferior, temporal, and nasal) and in the central zone and total and FAZ parameters (FAZ area, perimeter, and circularity) were analyzed and noted. Foveal avascular zone measurements were based on Angio Retina scans. Measurements were performed automatically, but curvature and strength sliders allowed for manual adjustment of the automatic FAZ area detection. Figure 1 and Figure 2 present the measurement details. Signal quality up to 10 was assessed by the tool for all analyses. Analyses with signal quality 8 and above were used for this study.

All participants’ RNFL thickness measurements were obtained through EDI OCT (Cirrus, Carl Zeiss Meditec, Dublin, CA, USA). RNFL thickness was measured according to the “Optic Disc Cube 200*200” method. The signal quality of the images had to be 7/10 or better to be included in this study.

The data was statistically evaluated using IBM SPSS Statistics Software (Version 23.0). Student’s *t*-test was used for mean age comparison between groups. Chi square tests were performed to compare categorical variables between the patient and control groups (sex and presence of optic neuritis). A linear mixed model was used for the comparison between BCVA, OCT-A parameters, and RNFL thickness in the patient and control groups and for the comparison of OCT-A parameters and RNFL thicknesses between PPMS-ON, PPMS+ON, and healthy eyes. Univariate tests and then pairwise comparisons with Bonferroni correction were performed between the groups. Any *p* value less than 0.05 was considered significant. Correlations between OCT-A parameters and disease duration, BCVA, RNFL thickness, and number of infusions were assessed using Pearson’s correlation test.

## 3. Results

A total of 38 PPMS patients receiving Ocrelizumab treatment and 31 healthy individuals were included in this study. The mean time since the last ON attack was 20.4 ± 4.75 months. Out of 43 PPMS patients, 5 were excluded due to low signal quality in the OCT-A and OCT measurements. The mean signal qualities were 9.82 ± 0.50 and 9.74 ± 0.44 in the patient and control groups, respectively. The segmentation of layers and FAZ areas was controlled, and automatically measured OCT-A parameters were used. Manual changes were not performed on these images. Lesions in the periventricular, cervical, and spinal cord were observed through magnetic resonance imaging. Table 1 displays the characteristics of the patient and control groups. Bilateral previous optic neuritis was observed in 18 patients, while unilateral ON was seen in 2 patients. The mean number of Ocrelizumab therapy sessions was 5.52 ± 2.26, and the mean duration of the disease was 9.19 ± 6.30 years in the patient group.

Table 2 shows a comparison between groups in terms of mean RNFL, superficial and deep vessel area densities, and FAZ parameters. The BCVA, RNFL thickness, and all parts of superficial vessel area densities except the central part between groups were statistically significantly different. Regarding the deep vessel area densities, the central and inferior parts differed between groups. The mean FAZ area was also statistically different between groups.

Table 3 displays a comparison of the mean RNFL, BCVA, and macular vessel area densities between the subgroups: the PPMS+ON, PPMS-ON, and control groups. Mean BCVA and RNFL were statistically different between all groups. Mean nasal, temporal, inferior part, and total superficial vessel area densities were statistically different between groups. The central and inferior parts of the deep vessel area densities were statistically significantly different. Among the FAZ parameters, the mean FAZ area was statistically different between the subgroups.

Pairwise comparisons with Bonferroni correction were conducted to identify the sources of statistical differences between the subgroups, and the results are presented in Table 4. While a statistically significant difference was found in the temporal part of the superficial vessel area density between the three groups, no statistically significant difference was found in the pairwise comparisons after a Bonferroni correction.

Correlation analyses were performed between OCT-A parameters (eyes with and without a previous ON history) and mean BCVA, RNFL thickness, disease duration, and number of therapy infusions. The results are provided as Supplementary Materials. No statistically significant correlation was found between the OCT-A parameters and mean BCVA, RNFL thickness, and disease duration in eyes with and without a previous ON history. The number of therapy infusions was negatively correlated with the central, inferior, and total superficial vessel area densities; nasal, central, temporal, inferior, and temporal deep vessel area densities; and the FAZ perimeter. The number of therapy infusions was also positively correlated with the FAZ area in the PPMS+ON group. In the PPMS-ON group, the central part in both the deep and superficial vessel area densities and the FAZ perimeter were negatively correlated, while the FAZ area was positively correlated with the number of therapy infusions.

## 4. Discussion

In the current study, PPMS patients with or without ON receiving the same treatment were investigated and compared with healthy controls. The mean BCVA was significantly different between patients and healthy controls. It is already known that optic neuritis is a common, frequently presenting manifestation, but visual deficits and structural loss of retinal axonal and neuronal integrity can occur even without a history of optic neuritis [13,14].

In the current study, the mean RNFL thickness gradually tapered in the subgroups, i.e., healthy control, PPMS-ON, and PPMS+ON eyes. These results are consistent with those in the literature. Albrecht et al. found a significant RNFL thickness in PPMS patients compared with that in healthy controls [15]. Miscioscia et al. performed a longitudinal study on PPMS patients (without ON) under Ocrelizumab treatment and found that the RNFL thickness was thinner compared with that in healthy controls [3]. Moreover, faster RNFL thickness was indicated in PMS patients without ON [3,16]. Poretto et al. found that the RNFL thickness was thinner in PPMS patients compared with that in healthy controls [17]. Saxena et al. reported that the temporal quadrant thickness seemed to be more affected in patients with MS-ON [18].

Except for the central part, superficial vessel area density measurements were significantly lower in PPMS patients compared with that in healthy controls. According to the subgroup analysis (with or without ON, and in healthy controls), there were significant differences in the nasal, temporal, and inferior quadrants and in total. In the nasal and inferior quadrant, the SVA densities were lower in eyes with and without ON compared with healthy eyes. Only eyes with previous ON had lower total SVA density than healthy eyes. Murphy et al. studied RRMS patients and found that superficial vessel density was reduced in RRMS patients compared with in healthy controls. Moreover, in RRMS patients with ON, superficial vessel density was lower compared with in patients without ON and in healthy controls [19]. Yılmaz et al. found a significant difference in terms of macular vessel densities of both the superficial and deep vascular plexus between RRMS patients and healthy controls [20]. Feucht et al. reported that eyes of RRMS patients with a history of ON had reduced vessel densities of the superficial and deep vascular plexuses compared with healthy controls [21]. The study by Jiang et al. revealed that the volumetric vessel density of the macular superficial capillary plexus was significantly higher in RRMS+ON cases [22]. All these studies focused on the macular vascular plexus in RRMS patients, not in PPMS patients. No previous study has evaluated OCT-A findings in the primary progressive subtype of MS. In this study, macular SVA and DVA densities in PPMS patients were investigated for the first time.

Deep vessel area density measurements in the central and inferior parts were significantly lower in PPMS patients compared with in the healthy controls. According to the subgroup analysis (with or without ON), DVA densities in the central and inferior quadrants were different between the groups and lower in eyes with ON compared with in healthy eyes. Murphy et al. found no effect of RRMS on deep vascular plexus density [19]. Studies in the literature have revealed insignificant differences or opposite results in terms of vessel density in the macular deep capillary plexus between MS patients and healthy controls [23,24,25]. A recent review reported that MS+ON had significantly lower foveal DCP vessel density and higher parafoveal DCP vessel density compared with the healthy control group. Moreover, MS-ON cases had no significant difference from the healthy control group in the whole image, foveal, and parafoveal DCP vessel densities. Comparing MS+ON with MS-ON cases, the former only had significantly higher whole image macular DCP vessel density, and no significant difference was found in the fovea, parafovea, and quadrants [26]. In contrast to studies focusing on RRMS patients, this study on PPMS patients revealed a rarefaction of the deep vessel area density of the macula.

The FAZ area was statistically larger in PPMS patients receiving Ocrelizumab therapy in this cohort. In the subgroup analysis, a statistically significant difference was found between PPMS+ON patients and healthy controls in terms of FAZ area. Yilmaz et al. revealed no significant difference between MS+ON vs. MS-ON eyes in the FAZ area [20]. Szilasi et al. also did not find a significant difference in terms of FAZ area between RRMS patients without ON and healthy controls [27]. Yılmaz et al. also investigated FAZ perimeter and circularity parameters and found no significant difference between RRMS patients and healthy controls, similarly to the current study [20]. The type and sample sizes affected the results of these studies. The current study is the first to investigate FAZ area, circularity, and perimeter in PPMS patients and found a significant difference in FAZ area only in the PPMS+ON group.

To evaluate the relationship between disease duration, number of therapy infusions, BCVA, and RNFL thickness and macular vessel area densities, correlation analyses were performed. A mild negative correlation was present between the number of therapy infusions and the central part of SVA and DVA densities in both the PPMS+ON and PPMS-ON groups. While studies have reported the relationship between OCT parameters, visual functions, and disease activity in MS patients [28,29,30], no study has yet been conducted on PPMS patients. Further investigations should be performed to shed light on this important issue.

The limitations of this study are the small sample sizes of the groups and the retrospective manner of this study. An evaluation of the parapapillary margin with OCT-A can give us more information, but this important data was missing from our study. In addition, correlations with other neurological functional tests can highlight the importance of OCT-A during follow-up with PPMS patients. On the other hand, an investigation of macular vessel area densities in PPMS patients receiving the same therapy (Ocrelizumab treatment) is unique. Further longitudinal studies with more detailed analyses are therefore needed to better understand this progressive disease.

In conclusion, superficial vessel area density measurements were significantly lower (except in the central part) in PPMS patients compared with those in healthy controls. Deep vessel area density measurements in the central and inferior parts were significantly lower in PPMS patients compared with those in healthy controls. In the subgroup analyses, the SVA and DVA densities in different parts were affected in eyes with ON compared with those in healthy eyes. The FAZ area was statistically larger in the PPMS patients. In addition, the FAZ area was larger in the PPMS+ON patients than that in the healthy controls. Macular superficial and deep vascular densities seem to be affected in PPMS patients, and an effect of previous optic neuritis was observed.

## Figures and Tables

**Table 1:** General characteristics of the patient and control groups.

	Patient Group *n*: 38	Control Group *n*: 31	*p*
Age (mean ± SD)	43.86 ± 8.81	43.67 ± 10.31	0.934
Disease duration (years, mean ± SD)	9.19 ± 6.26	0	
Gender (*n* %)			
Female	31 (81.6%)	22 (70.9%)	0.299
Male	7 (18.4%)	9 (29.0%)	
Attack (*n* %, eyes)			<0.001
ON (+)	38 (50%)	0	
ON (−)	38 (50%)	31 (100%)	

ON: previous optic neuritis history. SD: standard deviation.

**Table 2:** A comparison of macular vascular indexes between groups.

	Patient Group Mean ± SD 76 Eyes, 38 Patients	Control Group Mean ± SD 31 Eyes, 31 Patients	*p*
BCVA (decimal)	0.86 ± 0.22	1	0.003
RNFL thickness (μm)	82.38 ± 12.42	95.16 ± 8.86	<0.001
Superficial vessel area density			
Nasal	38.08 ± 2.67	39.71 ± 1.42	0.001
Central	15.95 ± 4.45	17.37 ± 3.26	0.146
Temporal	37.38 ± 2.82	38.90 ± 1.96	0.005
Superior	38.97 ± 2.66	40.19 ± 1.72	0.048
Inferior	38.42 ± 2.71	40.37 ± 1.78	0.01
Total	37.61 ± 1.98	38.57 ± 1.19	0.009
Deep vessel area density			
Nasal	43.09 ± 2.14	43.65 ± 1.43	0.329
Central	29.72 ± 5.13	33.07 ± 3.33	0.008
Temporal	43.00 ± 2.43	43.86 ± 0.92	0.140
Superior	43.40 ± 1.45	43.71 ± 0.88	0.295
Inferior	42.94 ± 2.10	43.90 ± 0.86	0.028
Total	42.32 ± 1.47	42.90 ± 0.79	0.142
Foveal avascular zone			
FAZ area	0.38 ± 0.11	0.32 ± 0.09	0.038
FAZ perimeter	3.04 ± 0.84	2.75 ± 0.70	0.402
FAZ circularity	0.57 ± 0.12	0.53 ± 0.09	0.079

BCVA: best-corrected visual acuity. RNFL: retinal nerve fiber layer. FAZ: foveal avascular zone.

**Table 3:** Comparison of macular vessel area densities and characteristics between subgroups.

	PPMS+ON Group (*n*: 38)	PPMS-ON Group (*n*: 38)	Control Group (*n*: 31)	*p*
BCVA	0.80 ± 0.27	0.92 ± 0.14	1	<0.001
Mean RNFL thickness	75.47 ± 9.96	89.28 ± 10.73	95.16 ± 8.86	<0.001
Superficial vessel area density				
Nasal quadrant	38.21 ± 2.41	37.95 ± 2.92	39.71 ± 1.42	0.005
Central	16.63 ± 4.14	15.27 ± 4.69	17.37 ± 3.26	0.342
Temporal quadrant	37.45 ± 2.36	37.31 ± 3.25	38.90 ± 1.96	0.019
Superior quadrant	38.84 ± 2.38	39.09 ± 2.94	40.19 ± 1.72	0.109
Inferior quadrant	38.20 ± 2.86	38.64 ± 2.58	40.37 ± 1.78	0.002
Total	37.4 ± 2.05	37.83 ± 1.92	38.57 ± 1.19	0.018
Deep vessel area density				
Nasal quadrant	42.91 ± 2.38	43.27 ± 1.89	43.65 ± 1.43	0.495
Central	29.50 ± 4.23	29.94 ± 5.95	33.07 ± 3.33	0.025
Temporal quadrant	43.04 ± 1.86	42.96 ± 2.92	43.86 ± 0.92	0.332
Superior quadrant	43.16 ± 1.46	43.65 ± 1.42	43.71 ± 0.88	0.173
Inferior quadrant	42.62 ± 2.47	43.26 ± 1.62	43.90 ± 0.86	0.035
Total	42.20 ± 1.53	42.44 ± 1.42	42.90 ± 0.79	0.250
Foveal avascular zone				
FAZ area	0.40 ± 0.12	0.36 ± 0.10	0.32 ± 0.09	0.034
FAZ perimeter	2.99 ± 0.76	3.08 ± 0.91	2.75 ± 0.70	0.699
FAZ circularity	0.58 ± 0.13	0.56 ± 0.12	0.53 ± 0.09	0.170

BCVA: best-corrected visual acuity. RNFL: retinal nerve fiber layer. FAZ: foveal avascular zone. PPMS+ON: primary progressive multiple sclerosis patient group with optic neuritis history. PPMS-ON: primary progressive multiple sclerosis patient group without optic neuritis history.

**Table 4:** Pairwise comparisons with Bonferroni corrections between the three subgroups.

	*p*/MD/95%CI PPMS+ON vs. PPMS-ON Groups			*p*/MD/95%CI PPMS+ON vs. Control Groups			*p*/MD/95%CI PPMS-ON vs. Control Groups		
	*p*	MD	95% CI	*p*	MD	95% CI	*p*	MD	95% CI
Mean RNFL thickness (μm)	<**0.001**	−15.65	−22.2/−9.08	<**0.001**	−20.59	−27.09/−14.08	0.20	−4.93	−11.43/1.56
Superficial vessel area density									
Nasal quadrant	1.00	0.122	−1.39/1.64	**0.03**	−1.58	−3.08/−0.07	**0.02**	−1.70	−3.2/−0.2
Central	1.00	0.122	−2.39/2.63	0.65	−1.35	−4.02/1.30	0.53	−1.47	−4.14/1.18
Temporal quadrant	1.00	0.01	−1.69/1.71	0.08	−1.54	−3.22/0.14	0.08	−1.55	−3.23/0.13
Superior quadrant	1.00	−0.35	−1.87/1.15	0.07	−1.42	−2.93/0.08	0.26	−1.06	−2.57/0.44
Inferior quadrant	1.00	−0.58	−2.17/1.01	**0.002**	−2.25	−3.83/−0.67	**0.03**	−1.67	−3.25/−0.09
Total	0.64	−0.59	−1.77/0.59	**0.02**	−1.27	−2.43/−0.10	0.45	−0.67	−1.84/0.49
Deep vessel area density									
Nasal quadrant	1.00	−0.35	−1.63/0.93	0.46	−0.74	−1.63/0.93	1.00	−0.38	−1.65/0.87
Central	1.00	−0.97	−4.04/2.09	**0.009**	−3.82	−6.86/−0.78	0.07	−2.85	−5.89/0.18
Temporal quadrant	1.00	0.07	−1.27/1.41	0.39	−0.83	−2.16/0.50	0.30	−0.90	−2.23/0.43
Superior quadrant	0.37	−0.50	−1.23/0.29	0.27	−0.56	−1.36/0.24	1.00	−0.06	−0.86/0.74
Inferior quadrant	0.49	−0.65	−1.80/0.48	**0.02**	−1.29	−2.43/−0.15	0.52	−0.63	−1.78/0.50
Total	1.00	−0.27	−1.12/0.58	0.11	−0.72	−1.56/0.12	0.58	−0.44	−1.29/0.39
Foveal avascular zone									
FAZ area	0.36	0.04	−0.02/0.11	**0.02**	0.08	0.01/0.15	0.64	0.03	−0.03/0.10
FAZ perimeter	1.00	0.01	−0.51/0.53	0.50	0.29	−0.22/0.81	0.53	0.28	−0.23/0.80
FAZ circularity	1.00	0.01	−0.05/0.09	0.54	0.04	−0.03/0.11	1.00	0.02	−0.05/0.09

PPMS+ON: primary progressive multiple sclerosis patient group with optic neuritis history. PPMS-ON: primary progressive multiple sclerosis patient group without optic neuritis history. FAZ: foveal avascular zone. Bold highlight: statistically significant *p* values. MD: mean difference. CI: confidence interval.

## Data Availability

The data that support the findings of this study are available from the corresponding author, Burçin Çakır, upon reasonable request because of ethical issues.

## Supplementary Materials

The following supporting information can be downloaded at https://www.mdpi.com/article/10.3390/diagnostics16060936/s1, Table S1. The statistical correlations between OCT-A parameters and RNFL thickness. Table S2. The statistical correlations between OCT-A parameters and disease duration. Table S3. The statistical correlations between OCT-A parameters and number of Ocrelizumab therapy sessions. Table S4. The statistical correlations between OCT-A parameters and BCVA.
