# Supplementary material for: Optical Coherence Tomography Angiography Findings in Primary Progressive Multiple Sclerosis Patients Receiving Ocrelizumab Treatment
Source: Diagnostics (Basel). 2026 Mar 22;16(6):936. doi: 10.3390/diagnostics16060936 (PMC13025269; doi:10.3390/diagnostics16060936)
Supplement: Supplementary file 1 [file diagnostics-16-00936-s001.zip › diagnostics-4121269-supplementary.pdf]

Supplementary material

Table S1: The statistical correlations between OCT-A parameters and RNFL thickness

	PPMS+ON Group(n:38) r (p)	PPMS-ON Group (n:38) r (p)	Control Group (n:31) r (p)
Superficial vessel area density	-0.106 (0.525)	0.175 (0.293)	0.067 (0.720)
Nasal Quadrant	0.033 (0.846)	0.014 (0.932)	-0.066 (0.725)
Central	-0.165 (0.322)	0.244 (0.140)	0.154 (0.409)
Temporal Quadrant	-0.032 (0.848)	0.248 (0.133)	-0.139 (0.457)
Superior Quadrant	0.077 (0.645)	0.165 (0.323)	0.083 (0.657)
Inferior Quadrant	0.002 (0.992)	-0.043 (0.797)	0.075 (0.689)
Total	0.155 (0.353)	0.133 (0.427)	-0.031 (0.870)
Deep vessel area density	0.254 (0.124)	0.153 (0.360)	-0.114 (0.541)
Nasal Quadrant	0.103 (0.537)	0.220 (0.184)	-0.179 (0.334)
Central	0.063 (0.708)	0.090 (0.592)	0.166 (0.372)
Temporal Quadrant	0.217 (0.191)	-0.055 (0.743)	0.029 (0.877)
Superior Quadrant	0.083 (0.619)	-0.001 (0.996)	-0.108 (0.564)
Inferior Quadrant	-0.139 (0.406)	-0.087 (0.605)	0.247 (0.180)
Total	0.151 (0.364)	0.007 (0.965)	-0.036 (0.848)
Foveal avascular zone	-0.161 (0.333)	-0.090 (0.592)	0.011 (0.952)
FAZ area	-0.106 (0.525)	0.175 (0.293)	0.067 (0.720)
FAZ perimeter	0.033 (0.846)	0.014 (0.932)	-0.066 (0.725)
FAZ circularity	-0.165 (0.322)	0.244 (0.140)	0.154 (0.409)

OCT-A: optical coherence tomography angiography RNFL: retina nerve fiber layer FAZ: foveal avascular zone, PPMS+ON: primary progressive multiple sclerosis with previous optic neuritis PPMS-ON: primary progressive multiple sclerosis without previous optic neuritis

Table S2: The statistical correlations between OCT-A parameters and disease duration

	PPMS+ON Group(n:38) r (p)	PPMS-ON Group (n:38) r (p)
Superficial vessel area density	-0.078 (0.643)	-0.044 (0.792)
Nasal Quadrant	0.031 (0.855)	0.206 (0.215)
Central	-0.083 (0.621)	0.122 (0.464)
Temporal Quadrant	0.114 (0.494)	0.134 (0.422)
Superior Quadrant	0.003 (0.986)	0.171 (0.306)
Inferior Quadrant	0.083 (0.620)	0.008 (0.961)
Total	-0.014 (0.935)	-0.058 (0.727)
Deep vessel area density	-0.061 (0.716)	-0.051 (0.761)
Nasal Quadrant	0.003 (0.986)	-0.102 (0.543)
Central	0.112 (0.504)	0.100 (0.552)
Temporal Quadrant	-0.083 (0.618)	0.009 (0.960)
Superior Quadrant	0.066 (0.694)	-0.059 (0.724)
Inferior Quadrant	0.005 (0.978)	-0.154 (0.357)
Total	-0.113 (0.501)	0.040 (0.811)
Foveal avascular zone	0.099 (0.554)	-0.171 (0.306)
FAZ area	-0.078 (0.643)	-0.044 (0.792)
FAZ perimeter	0.031 (0.855)	0.206 (0.215)
FAZ circularity	-0.083 (0.621)	0.122 (0.464)

OCT-A: optical coherence tomography angiography FAZ: foveal avascular zone, PPMS+ON: primary progressive multiple sclerosis with previous optic neuritis PPMS-ON: primary progressive multiple sclerosis without previous optic neuritis

Table S3: The statistical correlations between OCT-A parameters and number of ocrelizumab therapy

	PPMS+ON Group(n:38)<br>r (p)	PPMS-ON Group (n:38)<br>r (p)
Superficial vessel area density	-0.233 (0.160)	-0.213 (0.200)
Nasal Quadrant	**-0.413 (0.010)**	**-0.436 (0.006)**
Central	**-0.462 (0.004)**	-0.304 (0.064)
Temporal Quadrant	-0.267 (0.104)	-0.160 (0.338)
Superior Quadrant	**-0.443 (0.005)**	-0.250 (0.130)
Inferior Quadrant	**-0.406 (0.011)**	0.061 (0.716)
Total	**-0.349 (0.032)**	-0.196 (0.239)
Deep vessel area density	**-0.460 (0.004)**	**-0.375 (0.020)**
Nasal Quadrant	**-0.385 (0.017)**	-0.156 (0.349)
Central	-0.185 (0.265)	-0.121 (0.468)
Temporal Quadrant	**-0.461 (0.004)**	-0.178 (0.285)
Superior Quadrant	**-0.398 (0.013)**	-0.132 (0.430)
Inferior Quadrant	**0.466 (0.003)**	**0.364 (0.025)**
Total	**-0.390 (0.015)**	**-0.525 (0.001)**
Foveal avascular zone	-0,315 (0,054)	0,043 (0,796)
FAZ area	-0.233 (0.160)	-0.213 (0.200)
FAZ perimeter	**-0.413 (0.010)**	**-0.436 (0.006)**
FAZ circularity	**-0.462 (0.004)**	-0.304 (0.064)

OCT-A: optical coherence tomography angiography FAZ: foveal avascular zone, PPMS+ON: primary progressive multiple sclerosis with previous optic neuritis PPMS-ON: primary progressive multiple sclerosis without previous optic neuritis

Table S4: The statistical correlations between OCT-A parameters and BCVA

	PPMS+ON Group(n:38)<br>r (p)	PPMS-ON Group (n:38)<br>r (p)
Superficial vessel area density	-0.078 (0.643)	-0.044 (0.792)
Nasal Quadrant	0.031 (0.855)	0.206 (0.215)
Central	-0.083 (0.621)	0.122 (0.464)
Temporal Quadrant	0.114 (0.494)	0.134 (0.422)
Superior Quadrant	0.003 (0.986)	0.171 (0.306)
Inferior Quadrant	0.083 (0.620)	0.008 (0.961)
Total	-0.014 (0.935)	-0.058 (0.727)
Deep vessel area density	-0.061 (0.716)	-0.051 (0.761)
Nasal Quadrant	0.003 (0.986)	-0.102 (0.543)
Central	0.112 (0.504)	0.100 (0.552)
Temporal Quadrant	-0.083 (0.618)	0.009 (0.960)
Superior Quadrant	0.066 (0.694)	-0.059 (0.724)
Inferior Quadrant	0.005 (0.978)	-0.154 (0.357)
Total	-0.113 (0.501)	0.040 (0.811)
Foveal avascular zone	0.099 (0.554)	-0.171 (0.306)
FAZ area	-0.078 (0.643)	-0.044 (0.792)
FAZ perimeter	0.031 (0.855)	0.206 (0.215)
FAZ circularity	-0.083 (0.621)	0.122 (0.464)

OCT-A: optical coherence tomography angiography FAZ: foveal avascular zone, PPMS+ON: primary progressive multiple sclerosis with previous optic neuritis PPMS-ON: primary progressive multiple sclerosis without previous optic neuritis BCVA: best corrected visual acuity
